# Immune Response to *Lactobacillus plantarum* Expressing *Borrelia burgdorferi* OspA Is Modulated by the Lipid Modification of the Antigen

**DOI:** 10.1371/journal.pone.0011199

**Published:** 2010-06-18

**Authors:** Beatriz del Rio, Jos F. M. L. Seegers, Maria Gomes-Solecki

**Affiliations:** 1 Department of Molecular Sciences, University of Tennessee Health Science Center, Memphis, Tennessee, United States of America; 2 Lactrys Biopharmaceuticals BV, Leiden, The Netherlands; 3 Biopeptides Corp., Valhalla, New York, and Memphis, Tennessee, United States of America; CNRS/Université de Toulouse, France

## Abstract

**Background:**

Over the past decade there has been increasing interest in the use of lactic acid bacteria as mucosal delivery vehicles for vaccine antigens, microbicides and therapeutics. We investigated the mechanism by which a mucosal vaccine based in recombinant lactic acid bacteria breaks the immunological tolerance of the gut in order to elicit a protective immune response.

**Methodology/Principal Findings:**

We analyzed how the lipid modification of OspA affects the localization of the antigen in our delivery vehicle using a number of biochemistry techniques. Furthermore, we examined how OspA-expressing *L. plantarum* breaks the oral tolerance of the gut by stimulating human intestinal epithelial cells, peripheral blood mononuclear cells and monocyte derived dendritic cells and measuring cytokine production. We show that the leader peptide of OspA targets the protein to the cell envelope of *L. plantarum*, and it is responsible for protein export across the membrane. Mutation of the lipidation site in OspA redirects protein localization within the cell envelope. Further, we show that lipidated-OspA-expressing *L. plantarum* does not induce secretion of the pro-inflammatory cytokine IL-8 by intestinal epithelial cells. In addition, it breaks oral tolerance of the gut via Th1/Th2 cell mediated immunity, as shown by the production of pro- and anti-inflammatory cytokines by human dendritic cells, and by the production of IgG2a and IgG1 antibodies, respectively.

**Conclusions/Significance:**

Lipid modification of OspA expressed in *L. plantarum* modulates the immune response to this antigen through a Th1/Th2 immune response.

## Introduction

Over the past decade there has been increasing interest in the use of lactic acid bacteria (LAB) as mucosal delivery vehicles for vaccine antigens, microbicides and therapeutics [1]. A number of studies of oral vaccines generated from genetically engineered pathogenic or commensal bacteria have been reported [2], [3], [4], [5], [6], [7], [8], [9], [10], [11]. In humans, *Lactobacillus plantarum* belongs to the natural flora of the vagina and the gastrointestinal tract. We have recently developed an oral vaccine based in *L. plantarum* expressing the outer surface protein A (OspA), a lipoprotein from *Borrelia burgdorferi* [12].

Evidence suggests that the peptidoglycan layer of some LAB promote natural immuno-adjuvanticity [13], [14], [15], [16] and antigen localization on the cell wall makes it more accessible to the immune system as compared to intracellular or secreted proteins [17], [18]. Leader peptides mark proteins for translocation across the cytoplasmic membrane and lipid modification is of major importance both for anchoring exported proteins to the membrane and for protein function [19]. It has been shown that lipidation at the first aminoacid of the mature OspA protein is essential to induce an immune response [20], [21]. In this study, we examined the influence of post-translational processing in the localization of OspA lipoprotein on the cell envelope of *L. plantarum.*

The normal immune response to harmless gut antigens and commensal bacteria is the induction of a local and systemic immunological tolerance, known as oral tolerance [22], [23]. Immunological tolerance can be exploited to develop immunotherapies for autoimmune and inflammatory diseases but it is also the obstacle to the development of oral vaccines [23]. As a result of our observation that an oral vaccine based in *L. plantarum* expressing the OspA lipoprotein induced a protective, IgG-based, immune response in mice [12] we set out to further understand the processes that determine the immunological consequences of oral administration of antigen. We investigated the role of *L. plantarum* expressing OspA lipoprotein in breaking the oral tolerance of the gut and if this immune response was dependent on the lipid modification of OspA.

## Materials and Methods

### Ethics statement

The procedures involving human blood were approved by the Institutional Review Board (IRB) of the University of Tennessee Health Science Center, under the provisions of the Department of Health and Human Services Regulations for Protection of Human Subjects (45 CFR 46) and similar FDA Regulations (21 CFR 50 and 56). All participants provided written informed consent. The procedures involving mice were in accordance with the Animal Welfare Act and the Public Health Service Policy on Humane Care and Use of Laboratory Animals and were approved by the Institutional Animal Care and Use Committee (IACUC) at the University of Tennessee Health Science Center.

### Bacterial strains, cell lines and culture conditions

*L. plantarum* was grown at 30°C in LM medium [1% proteose peptone (w/v), 1% beef extract (w/v), 0.5% yeast extract (w/v), 0.5% lactose (w/v), 9 mM ammonium citrate, 61 mM sodium acetate anhydrous, 0.4 mM magnesium sulfate, 0.3 mM manganese sulfate, 11.2 mM dipotassium phosphate, 0.5% Tween 20 (v/v)], supplemented with 10 µg/ml of chloramphenicol (Cm). *E. coli* was grown at 37°C in TBY medium (BD, Franklin Lakes, NJ). T84 human colonic carcinoma epithelial cells were obtained from the American Type Culture Collection (ATCC, CCL-248, Manassas, VA). T84 cells were maintained at 37°C, 5% CO_2_ in DMEM-F12K medium modified by ATCC, containing 10% FCS, 100 U/ml penicillin and 100 µg/ml streptomycin.

### Plasmid construction and characterization of expressed antigens

A vector that expresses the full-length *ospA* gene from *B. burgdogferi* in *L. plantarum* was previously described [12]. To generate a vector expressing the OspA mutant, we designed primers in which we replaced the codon corresponding to Cys^17^ with Asp^17^. Expression vectors were then transformed into *L. plantarum* strain 256 to obtain the clones LpA and LpA_D17_, respectively. Protein expression was checked by immunoblot as follows. Recombinant *L. plantarum* cells were disrupted with a French® press (Thermo Electron Corporation, Milford, MA), supernatants were analyzed on a 12% denaturing polyacrilamide gels and electrotransferred to a polyvinylidene difluoride membrane (PVDF, Millipore, Billerica, MA) for analysis with an OspA-specific monoclonal antibody (mAb 184.1). To evaluate export and lipidation of OspA and its mutant expressed by recombinant *Lactobacillus*, we performed Triton X-114 phase partitioning [24]. *L. plantarum* cultures were grown overnight at 30°C, harvested and resuspended to an OD_600_ of 1.0 in PBS. Bacteria were disrupted with a French® press and the insoluble material (membrane and cell wall) was separated from the cytosol fraction by centrifugation. This cell envelope fraction was suspended in 1 ml of ice-cold 2% Triton-X114 (v/v) in PBS. The fractions were rotated end over end at 4°C for 1 h and were phase-separated by warming the solution for 30 min in a water bath at 37°C followed by centrifugation for 15 min at 25°C. The separated detergent and aqueous phases were each washed three times. The solutions were then rewarmed and recentrifuged as described and the detergent and aqueous phases were collected. Ten (10) µl of each phase was analyzed on 15% denaturing polyacrylamide gels, electrotransferred to PVDF filters, and used for immunoblot analysis. OspA-specific monoclonal antibody LA2.2 (1∶100) was used as primary antibody, goat anti-mouse IgG (H+L) conjugated to alkaline phosphatase (1∶1000; Pierce Rockford, IL) was used as secondary antibody and the immunoblot was developed by BCIP/NBT™ (KPL, Washington, DC). The protein bands corresponding to each OspA antigen were quantified by densitometry using a Multi Image™ Light Cabinet and the AlphaEase™ software (Alpha Innotech Corporation, San Leandro, CA). The results were plotted as a percentage of the total OspA content for each recombinant *Lactobacillus*.

A vector that expresses the full-length *ospA* gene from *B. burgdogferi* in *Escherichia coli* (EcA) was previously described [25]. To generate the vector pET9c-_mut_OspA that expresses the OspA mutant (_mut_OspA), we designed primers to amplify the *B. burgdorferi ospA* gene lacking the signal sequence and we cloned the PCR fragment into pET9c. The expression vectors were transformed into *E. coli* BL21 (DE3) pLysS, the recombinant proteins were purified and the endotoxines were removed using the Detoxi-Gel™ Endotoxin Removing Gel (Pierce Biotechnology, Rockford, IL).

### Cell fractionation assay

*Lactobacillus* cultures were grown overnight at 30°C, harvested and resuspended to an OD_600_ of 1.0 in TGE buffer [25 mM Tris-HCl pH 8, 50 mM glucose, 10 mM EDTA pH 8]. For cell wall removal, aliquots of 1 ml were treated with 250 KU/ml of lysozyme (Lyz; Novagen, Gibbstown, NJ) for 45 min at 37°C. Protoplasts and cell wall fractions were separated by centrifugation and the supernatants containing the cell walls collected. Protoplasts were washed, resuspended in TED buffer [25 mM Tris-HCl pH 8, 1 mM EDTA, 1 mM DTT] and disrupted with a French® press. Supernatants were ultracentrifugated for 1 h to separate membranes from cytosol fractions. Three (3) µg of each fraction was analyzed on 15% denaturing polyacrylamide gels, electrotransferred to PVDF filters and used for immunoblot analysis with mAb LA2.2 as described above.

### Indirect immunofluorescence microscopy

Recombinant *Lactobacillus* were treated with and without 250 kU/ml of Lyz in TGF buffer [100 mM Tris-HCl pH.8, 50 mM glucose, 1% FBS (v/v) (Hyclone, South Logan, UT)] for 30 min. Cells were washed and resuspended in TGF buffer with mAb LA2.2 (1∶100) for 1 h at room temperature, washed three times with 500 µl TGF buffer and resuspended on 100 µl of the same buffer. Aliquots of 10 µl were placed on slides and air-dried at 37°C for 1 h. Slides were incubated with Alexa Fluor 488-labeled goat anti-mouse IgG antibody (1∶250) (Molecular Probes, Invitrogen, Carlsbad, CA) in 100 µl TGF buffer at 23°C for 1 h with intermittent gentle mixing. After incubation, slides were washed three times with TGF buffer and fixed with 4% PBS–buffered formaldehyde (methanol free; Ted Pella Inc., Redding, CA) for an additional 15 min at room temperature. Labeled cells were mounted in VectaShield medium containing 4,6-diamidino-2-phenylindole (DAPI; Vector Laboratories, Burlingame, CA) and visualized using a Zeiss inverted Axiovert 200 motorized microscope and analyzed using the Axiovision 4.3 software.

### Live-cell ELISA (lcELISA)

To further investigate the localization of antigens on the *Lactobacillus* cell envelope, we developed an indirect live-cell enzyme-linked immunosorbent assay (lcELISA). *Lactobacillus* cultures were grown overnight at 30°C, harvested and resuspended to an OD_600_ of 1.0 in TG buffer [100 mM Tris-HCl pH.8, 50 mM glucose]. For cell wall digestion, 1 ml aliquots were resuspended in TG buffer with or without Lyz (250 kU/ml) for 5 or 45 min at 37°C. Cells were washed twice with TG buffer, resuspended in the same buffer supplemented with 3% BSA (Bovine Serum Albumin, Sigma), and incubated with mAb LA2.2 (1∶500). Samples were washed twice and incubated for 30 min with goat anti-mouse IgG (H+L) antibodies conjugated to alkaline phosphatase (1∶1000). After an extensive wash, labeled cells were incubated with *p*NPP Microwell Substrate System (KPL). Microtiter plates were loaded with 100 µl of each cellular suspension, and optical densities were measured at 405 nm by a Spectra MAX plus ELISA reader (Molecular Devices, Sunnyvale, CA).

### IL-8 production by human epithelial cells

T84 cells (human colon carcinoma epithelial cell line) were seeded in 24-well tissue culture plates (BD Biosciences, San Jose, CA) at a density of 1×10^6^ cells/well and grown until they reached 90 to 95% confluence. *L. plantarum* cells were killed by exposure to UV light for 1 h and the lack of cell viability was confirmed by culture in MRS agar. T84 cells were co-cultured with UV-killed recombinant *L. plantarum* at a MOI 10∶1 bacteria per cell, for 48 h. *L. plantarum* control and 0.5 µg/ml TNFα were used as negative and positive controls, respectively. Supernatants were collected and the human IL-8 production was measured by ELISA (Quantikine, R&D Systems, Minneapolis, MN).

### Isolation of human Peripheral Blood Mononuclear Cells and monocytes

Human peripheral blood was collected into heparin vacutainer tubes (BD Bioscience, Franklin Lakes, NJ) from healthy individuals. Peripheral blood mononuclear cells (PBMCs) were isolated by Ficoll-Paque density gradient centrifugation (GE Healthcare, Uppsala, Sweden). A final suspension was made in complete RPMI 1640 (Hyclone), supplemented with 10% [v/v] FBS, 100 U/ml penicillin, 100 µg/ml streptomycin, 0.25 µg/ml and fungizone. Cell viability (greater than 95%) was determined by trypan blue exclusion. Human monocytes were purified from PBMC using the Monocyte Isolation Kit II (Miltenyi Biotec, Auburn, CA), according to the manufacturer's recommendations. These monocytes were then used to produce Monocyte Derived Dendric Cells (MDDC) as described below.

### Stimulation for cytokine production

To derive dendritic cells from PBMCs and monocytes we cultured 1×10^6^ or 2×10^5^ cells/well, respectively, in 24-well tissue culture plates for 5 days in 2 ml of complete RPMI 1640 supplemented with 10 ng/ml IL-4, and 100 ng/ml recombinant human granulocyte-macrophage colony-stimulating factor (GM-CSF) (R&D system, Minneapolis, MN). Cultures were placed at 37°C in a 5% CO_2_ humidified incubator. Every two days the medium was removed and 2 ml of fresh complete medium was added. At day 5 the cells were co-cultured with 2.5 µg/ml of purified recombinant OspA or UV-killed recombinant *Lactobacillus* at MOI 10∶1 colony-forming units per cell for 48 h at 37°C. 100 ng/ml of lipopolysaccharide (LPS) from *Escherichia coli* O111:B4 (LIST Biological Laboratories, Campbell, CA) and *L. plantarum* were used as positive and negative control, respectively. Supernatants were collected and TNFα, IL-12, IFNγ, IL-6, and IL-10 were quantified by ELISA (Quantikine, R&D Systems).

### Intragastric inoculation of recombinant *L. plantarum*

*L. plantarum* expressing the target antigen was cultured in LM medium supplemented with 10 µg/ml Cm, and grown at 30°C to an OD_600_ of 1.0. That is the equivalent of 1×10^9^ cells/ml corresponding to approximately 125 µg of total protein. The cells were harvested by centrifugation at 3000 *g* for 10 min at 4°C and resuspended in 20% glycerol/phosphate buffered salt solution (Gibco, Grand Island, NY) in 1% of the initial volume. Cell suspensions in aliquots of 2 ml were frozen quickly in a dry ice bath and stored at −80°C. Aliquots were thawed at 4°C and 400 µl (4×10^10^ cells) were placed in a ball-tipped syringe for oral gavage inoculation. Groups of four female C3H-HeJ mice (6–8 weeks old, Charles River, Boston, MA) were immunized by intragastric inoculation of 4×10^10^ *Lactobacillus* expressing OspA recombinant antigens. *L. plantarum* (Lp) was used as control. Mice received the first immunization, twice daily, for 8 days (days 1–4 and 8–11). After resting for two weeks the mice were bled (day 28). On days 29–32 they received the 1^st^ oral boost and rested for an additional 2 weeks. On day 49, the mice were bled. On days 50–53 they received the 2^nd^ oral boost and rested for an additional 2 weeks. On day 68 mice were terminated, and blood, stool and bronchoalveolar lavage fluids (BAL) were collected. The animals were housed in a pathogen free colony on hardwood chip bedding in microisolator cages at the University of Tennessee Health Science Center, Memphis, TN. They were maintained on a 12 h light-dark cycle, in a room kept al 23°C with 50–60% relative humidity; they were given tap water and sterile irradiated rodent chow *ad libitum*.

### Humoral immune response

Serum, stool and BAL from orally inoculated mice were tested by indirect ELISA for the presence of total IgG, IgG1, IgG2a or IgA to OspA. Purified recombinant OspA was coated at 0.5 µg/ml on Nunc MaxiSorp™ flat-bottom ELISA plates (eBioscience, San Diego, CA) and indirect ELISA was performed using serum (1∶500), stool (1∶10) or bronchoalveolar fluid. Anti-mouse IgG, anti-mouse IgG1, anti-mouse IgG2a or anti-mouse IgA horseradish peroxidase-conjugated antibodies (1∶2,000) (Jackson ImmunoResearch, West Grove, PA) was used as secondary antibody.

### Statistical Analysis

All data is represented as mean ± standard deviation. Statistical analyses were performed using Student's *t*-test. *p*<0.05 are considered statistically significant.

## Results

### Construction of recombinant *L. plantarum* and evaluation of protein lipidation

**Figure 1:**
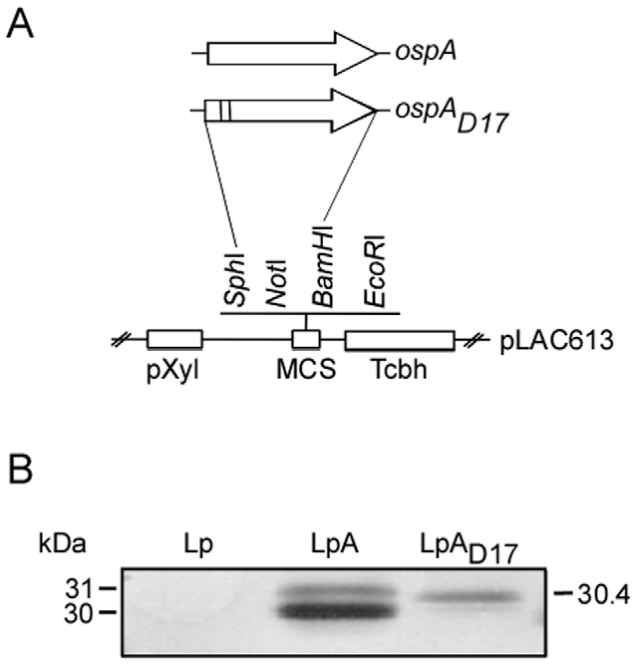
Characterization of recombinant *Lactobacillus*. Schematic representation of the *ospA* and *ospA_D17_* constructs (A), and immunoblot characterization (B). Whole-cell extract of control, OspA- and OspA_D17_-expressing *L. plantarum* (Lp, LpA, and LpA_D17_, respectively) were analyzed on a 12% SDS-PAGE, transferred to PVDF membrane and tested with OspA-specific monoclonal antibody 184.1. Legend: pXyl, xylose promoter; MCS, multicloning site; Tcbh, Rho independent transcriptional terminator.

Previously, we reported the cloning and expression of full length OspA from *B. burgdorferi* in a lactobacilli expression vector and designed an oral vaccine candidate for Lyme disease (LpA) [12]. For this study, we mutated the conserved lipidation motif of *ospA* by replacing the cystein at position 17 with aspartic acid to generate a protein that lacks the post-translational lipid attachment (Fig.1A). Total extracts of *L. plantarum* carrying the empty vector (Lp), and expressing OspA (LpA) or OspA_D17_ (LpA_D17_) were analyzed by SDS-PAGE and protein expression was confirmed using anti-OspA monoclonal antibody, mAb 184.1 (Fig.1B). The two bands seen for LpA may represent the lipidated unprocessed OspA protein (the leader peptide has not been cleaved, 31 kDa) and the lipidated mature protein (the leader peptide has been cleaved, 30 kDa). The single band seen for LpA_D17_ (30.4 kDa) may represent the unlipidated OspA (containing the leader peptide).

**Figure 2:**
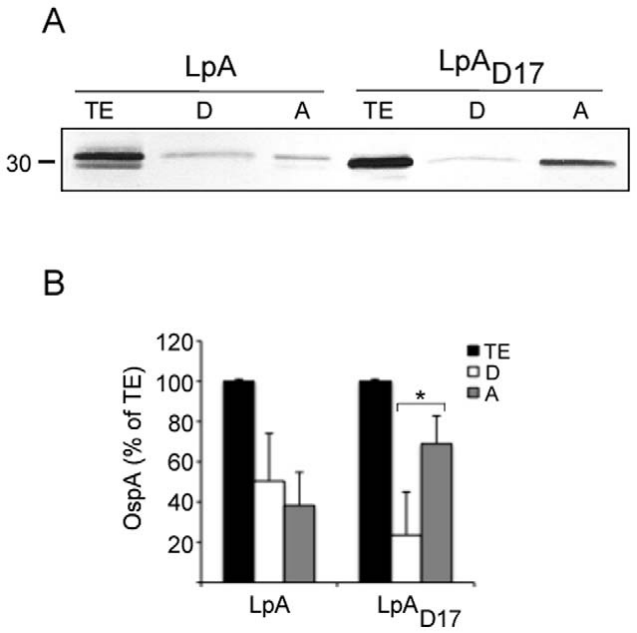
Evaluation of protein export and lipidation. OspA- and OspA_D17_-expressing *L. plantarum* were disrupted with a French® press, the insoluble material (cell envelope) was extracted with Triton X-114 and partitioned into detergent and aqueous phases. Protein fractions were analyzed on a SDS-PAGE and tested by immunoblot with OspA-specific monoclonal antibody LA2.2 (A). Protein was quantified by densitometry. The results were plotted as a percentage of the total OspA content for each recombinant *Lactobacillus* (B). TE, total extract; D, detergent phase; A, aqueous phase; OspA- and OspA_D17_-expressing *L. plantarum* (LpA and LpA_D17_, respectively). *p<0.05.

We further evaluated the export and lipidation of OspA and its mutant by Triton X-114 phase partitioning of the cell envelope (Fig. 2). In the cell envelope of *Lactobacillus*, OspA partitions to the detergent phase, suggesting that OspA is exported through the membrane and that the amount of OspA that is hydrophobic (∼50%) is higher than the hydrophilic form (∼40%), although not significantly so. In contrast, the mutant OspA_D17_ partitions mostly to the aqueous phase, suggesting that, in addition to being exported, the mutant generated more hydrophilic OspA (∼70%) than hydrophobic (∼25%) (Fig. 2A and 2B). If about 25% of OspA is associated with the cell envelope due to hydrophobicity of the leader sequence (i. e. OspA_D17_ in the detergent phase of LpA_D17_), then the difference (i. e. ∼30% additional wildtype OspA in the detergent phase of LpA) should be protein that is associated with the cell envelope via the lipid anchor, or the lipidated form of OspA. Differences for the mutant OspA_D17_ partitions are statistically significant (Fig. 2B, *p<0.05).

### Localization of recombinant antigens in *L. plantarum*

**Figure 3:**
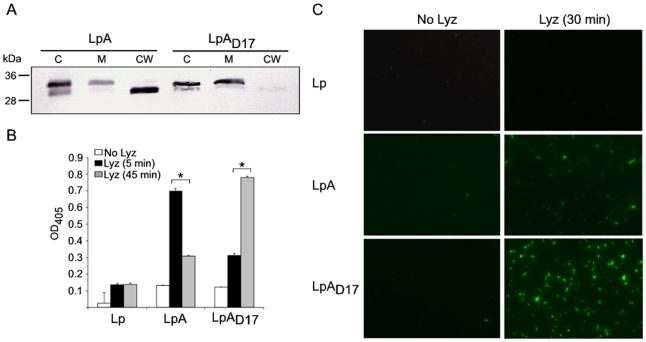
Localization of recombinant antigens in *L. plantarum*. Localization of the recombinant antigens was studied by (A) serial cell fractionation, (B) live-cell ELISA (lcELISA) and (C) Immunofluorescence Assay (IFA). (A) Immunoblot of cellular fractions of *L. plantarum* expressing OspA antigens: C, cytosol; M, membrane; CW, cell wall. *L. plantarum* was treated with 250 kU/ml Lysozyme (Lyz) for 45 min to digest the cell wall; protoplasts were disrupted and, membrane and cell wall fractions were separated by ultracentrifugation. 3 µg of each fraction was analyzed in a 12% SDS-PAGE, transferred to PVDF membrane, and tested by immunoblot with OspA-specific monoclonal antibody LA2.2 (mAb LA2.2). (B) Live recombinant *L. plantarum* were treated during 0, 5 or 45 min with Lyz and then subjected to lcELISA using mAb LA2.2 and anti-mouse IgG secondary antibody labeled with alkaline phosphatase. The Optical Density at 405 nm (OD_405_) of the mean endpoint titer was determined. The average of triplicate samples per sample was determined and the error bar indicates standard deviation. (C) Live recombinant *L. plantarum* were treated with or without Lyz for 30 min. After cell wall removal, the cells were incubated with mAb LA2.2 followed by Alexa Fluor 488-labeled goat anti-mouse IgG (1∶250) antibodies. Immunofluorescence staining was visualized using a Zeiss inverted Axiovert 200 microscope, and the images were acquired using AxioVision software. *p<0.001. Results are representative of one of three independent experiments.

Next, we performed serial fractionation experiments in order to determine where the recombinant proteins are localized within the cell envelope of *L. plantarum* (Fig. 3A). We treated recombinant *L. plantarum* with Lysozyme (Lyz) for 45 min and separated the cell wall fraction from the protoplast by centrifugation. We then disrupted the cells and separated the membrane from the cytosol fractions by ultracentifugation. The three fractions were then analyzed on denaturing polyacrylamide gels and OspA was identified by Immunoblot with anti-OspA monoclonal antibody (mAb LA2.2). Lipidated OspA expressed in *L. plantarum* is exported through the membrane and accumulates in the cell wall, while unlipidated OspA_D17_ remains mostly attached to the membrane (Fig. 3A). Next, we wanted to analyze the localization of the recombinant proteins on the surface of *L. plantarum.* We incubated live recombinant *L. plantarum* with and without Lyz and we performed both live-cell ELISA (lcELISA) and immunofluorescence (IFA) assays. For lcELISA, we incubated the recombinant *L. plantarum* with Lyz for 5 and 45 min, the cells were washed and incubated with mAb LA2.2 (Fig. 3B). For immunofluorescence, we performed a 30 min incubation with Lyz after which the cells were washed, incubated with mAb LA2.2 followed by Fluor 488-labeled goat anti-mouse IgG (1∶250). Staining was visualized using a Zeiss inverted Axiovert 200 microscope (Fig. 3C). In both assays, IFA and lcELISA, reactions without lyz (No Lyz) detect protein that is exposed on the surface of the cell. The epitope used to detect OspA and OspA_D17_ is located in the carboxyl terminal of the protein and is not surface exposed (Fig. 3B and 3C). Reactions with Lyz digest the peptidoglycan that releases the OspA that is trapped within the cell wall and expose OspA that is attached to the membrane (Lyz 5 min, 45 min or 30 min, Fig. 3B and 3C). Our results indicate that lipidated OspA (LpA) is trapped within the cell wall. That is, a 5 min digestion with Lyz exposes a high amount of OspA that is associated with the peptidoglycan layer (Fig. 3B) and a 45 min or 30 min digestion exposes a lower amount of OspA that is associated with the peptidoglycan layer (Fig. 3B and 3C), suggesting that OspA might not be attached to the membrane. In contrast, unlipidated OspA_D17_ (LpA_D17_) is trapped within the peptidoglycan layer of the cell wall, maybe attached to the membrane. That is, a 5 min digestion with Lyz exposes a low amount of OspA that is associated with the peptidoglycan layer (Fig. 3B) and a 45 min or 30 min digestion exposes a high amount of OspA that is associated with the peptidoglycan layer and the membrane (Fig. 3B and 3C).

### Cellular immune response to recombinant *L. plantarum*

**Figure 4:**
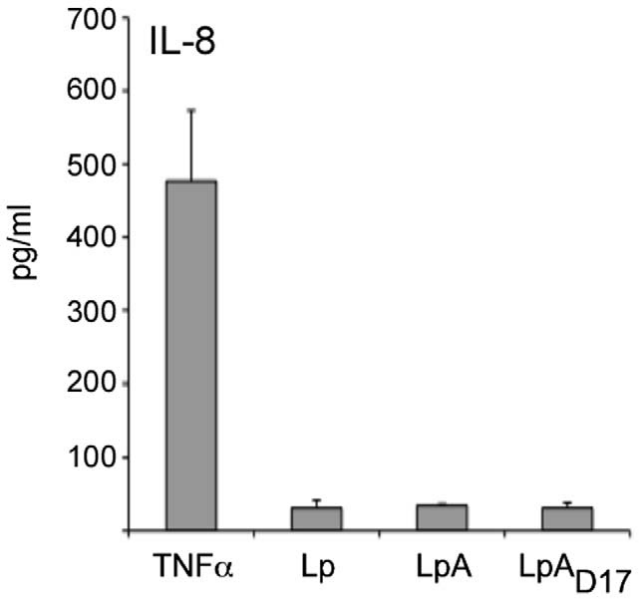
Production of IL-8 in human epithelial cells co-cultured with recombinant *L. plantarum*. Human epithelial cells (T84) were seeded in 24-well plates (10^6^ cells/well) and grown until they reached about 95% confluence. UV-killed *L. plantarum* expressing OspA (LpA) or the mutant OspA_D17_ (LpA_D17_) were co-cultured with T84 cells at MOI 10:1 colony-forming units per cell for 48 h and culture supernatants were collected to determine IL-8 secretion by sandwich ELISA (Quantikine). 0.5 µg/ml TNFα and UV-killed *L. plantarum* (Lp) were used as positive and negative control, respectively. The average of triplicate samples was determined and the error bar indicates standard deviation. Results are representative of one of three independent experiments.

In order to analyze the potential inflammatory response to the oral administration of *L. plantarum* expressing OspA lipoprotein, we performed an assay using monolayer cultures of intestinal epithelial cells (T84), a human colon carcinoma cell line, stimulated with UV-killed LpA and LpA_D17_ and determined the production of IL-8 (Fig. 4). The co-culture of T84 cells with UV-killed LpA or LpA_D17_ did not induce significant production of the pro-inflammatory chemokine IL-8 in comparison to the positive control (TNFα).

**Figure 5:**
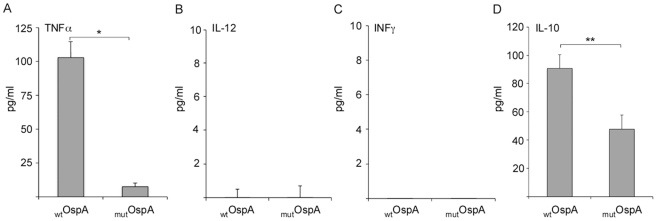
Production of cytokines in human MDDCs co-cultured with purified recombinant _wt_OspA or _mut_OspA. Monocytes were isolated from human PBMCs by MACS using the Monocyte Isolation Kit II. Monocytes were derived to immature DCs (MDDCs) by cultivating the cells in the presence of 100 nM GM-CSF and 10 nM IL-4 for 5 days. MDDCs (2×10^5^ cells/well) were seed in 24 well plates and co-cultured with 2.5 µg/ml of _wt_OspA or _mut_OspA. After 48 h of stimulation, supernatants were collected and TNFα (A), IL-12 (B), IFNγ (C) and IL-10 (D) cytokine production was measured by sandwich ELISA (Quantikine). *p<0.001,**p<0.05. Results are representative of one of three independent experiments.

Dendritic cells (DCs) play a decisive role in the innate and adaptive immune response, as they produce cytokines in response to antigen stimulation and activated T cells. Monocytes were isolated from human Peripheral Blood Mononuclear Cells (PBMCs) and were treated with GM-CSF and IL-4 to derive to MDDCs. Because OspA is known to trigger TLR2-based receptors we stimulated MDDCs with purified proteins in order to access the effect of the naked protein (wildtype OspA (_wt_OspA) versus a OspA mutant lacking the leader peptide (_mut_OspA)), on cytokine induction. We co-cultured MDDCs with 2.5 µg/ml of purified _wt_OspA or _mut_OspA during 48 h and the production of pro-inflammatory cytokines TNFα, IL-12, IFNγ, and anti-inflammatory cytokine IL-10 was quantified by ELISA (Fig. 5A, 5B, 5C and 5D, respectively). _wt_OspA induced TNFα and IL-10 production (Fig 5A and 5D) but it did not induce neither IL-12 nor IFNγ (Fig. 5B and 5C). Compared to _wt_OspA, the OspA mutant lacking the leader peptide, _mut_OspA, showed a 10 fold-decrease in the TNFα production (Fig. 5A) and a 2 fold-decrease in the IL-10 production (Fig. 5D). Similarly to _wt_OspA, _mut_OspA did not induce neither IL-12 nor IFNγ cytokine production (Fig. 5B and 5C). Differences between _wt_OspA and _mut_OspA are statistically significant (*p<0.001 and **p<0.05).

**Figure 6:**
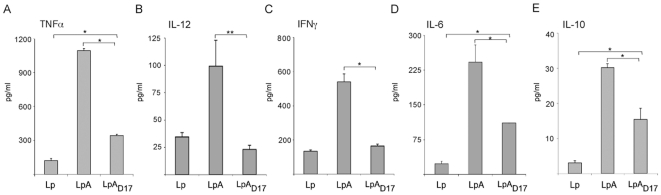
Production of cytokines in human PBMC/DCs co-cultured with recombinant *Lactobacillus*. Human Peripheral Blood Mononuclear Cells (PBMCs) were treated with 100 nM GM-CSF and 10 nM IL-4 during 5 days to derive monocytes into dendritic cells. 10^6^ cells/well were seed in 24 well plates and co-cultured with UV-killed recombinant *Lactobacillus* expressing OspA (LpA) or the mutant OspA_D17_ (LpA_D17_) at MOI 10:1 colony-forming units per cell. 100 ng/ml *Escherichia coli* O111:B4 lipopolysaccharide (LPS) and *L. plantarum* (Lp) were used as positive and negative control, respectively. After 48 h of stimulation, supernatants were collected and TNFα (A), IL-12 (B), IFNγ (C), IL-6 (D) and IL-10 (E) cytokine production was measured by sandwich ELISA (Quantikine). *p<0.001,**p<0.02. Results are representative of one of three independent experiments.

Next, we treated PBMCs with GM-CSF and IL-4 to derive the monocyte population into immature dentritic cells (PBMC/DCs), and we investigated the cytokine production of human PBMC/DCs co-cultured with UV-killed recombinant *L. plantarum* expressing either lipidated OspA (LpA), the mutant unlipidated OspA (LpA_D17_) or the control (Lp). The amount of pro-inflammatory cytokines TNFα, IL-12, IFNγ and IL-6, and anti-inflammatory cytokine IL-10 was quantified by ELISA (Fig. 6). As compared to the control, LpA induced TNFα by 9 fold, IL-12 by 3 fold, IFNγ by 4 fold and IL-6 by 10 fold (Fig. 6A, 6B, 6C and 6D). Additionally, anti-inflammatory cytokine IL-10 was 7 fold upregulated by stimulation with recombinant *L. plantarum* expressing lipidated (LpA) as compared to the control (Lp) (Fig. 6E). Compared to LpA, the mutant *L. plantarum* expressing the unlipidated OspA (LpA_D17_) induced significantly less TNFα, IL-12, IFNγ, IL-6 and IL-10 (Fig. 6A, 6B, 6C, 6D and 6E). Differences between LpA and LpA_D17_ are statistically significant (*p<0.001 and **p<0.02). Further, compared to the control (Lp), LpA_D17_ induced TNFα, IL-6 and IL-10 (*p*<0.001). LPS was used as a positive control and upregulated secretion of all cytokines tested (data not shown).

**Figure 7:**
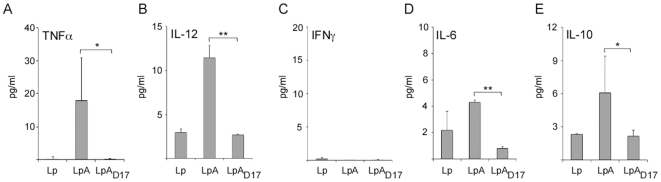
Production of cytokines in human MDDC co-cultured with recombinant *Lactobacillus*. Monocytes were isolated from human PBMCs by MACS using the Monocyte Isolation Kit II. Monocytes were derived to immature DCs (MDDCs) by cultivating the cells in the presence of 100 nM GM-CSF and 10 nM IL-4 for 5 days. MDDCs (2×10^5^ cells/well) were co-cultured with UV-killed recombinant *Lactobacillus* expressing OspA (LpA) or the mutant OspA_D17_ (LpA_D17_) at MOI 10:1 colony-forming units per DC. 100 ng/ml *Escherichia coli* O111:B4 lipopolysaccharide (LPS) and *L. plantarum* (Lp) were used as positive and negative control, respectively. After 48 h of stimulation, supernatants were collected and TNF (A), IL-12 (B), IFNγ (C), IL-6 (D) and IL-10 (E) cytokine production was measured by sandwich ELISA (Quantikine). *p<0.05,**p<0.001. Results are representative of one of three independent experiments.

In order to dissect these results, we further purified monocytes by MACS to derive MDDC and analyzed cytokine production after stimulation (Fig. 7). As expected, we determined that LpA induced higher production of proinflammatory cytokines TNFα (17 fold upregulated), IL-12 (∼4 fold upregulated) and IL-6 (1–2 fold upregulated) as compared to the control (Lp) (Fig. 7A, 7B and 7D, respectively). There was no production of IFNγ by monocyte derived dendritic cells (Fig. 7C). Compared to LpA, the mutant *L. plantarum* expressing the unlipidated OspA (LpA_D17_) abrogated this response as seen by the decreased production of TNFα, IL-12 and IL-6 (Fig. 7A, 7B and 7D). Additionally, IL-10 was ∼2–3 fold upregulated after stimulation with recombinant *L. plantarum* expressing lipidated (LpA) and the mutant appeared to impair this response (Fig. 7E). Differences between LpA and LpA_D17_ are statistically significant (*p<0.05 and **p<0.001).

### Antibody response to oral administration of recombinant *L. plantarum*

**Figure 8:**
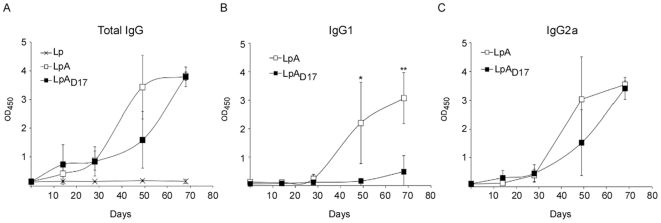
Antibody response to oral administration of recombinant *L. plantarum*: serological IgG. C3H-HeJ mice were inoculated intragastrically with *L. plantarum* expressing OspA (LpA) or the mutant OspA_D17_ (LpA_D17_). Control mice were inoculated with *L. plantarum* (Lp). Serum samples were collected at days 0, 14, 28, 49 and 68, and specific serological anti-OspA total IgG (A), IgG1 (B) and IgG2a (C) antibodies were measured by indirect ELISA. The results are expressed as Optical Density at 450 nm (OD_450_). The average of triplicate samples per mouse was determined and the error bar indicates standard deviation. *p<0.05, **p<0.01. Results are representative of one of three independent experiments. *n* = 4 mice per group.

**Figure 9:**
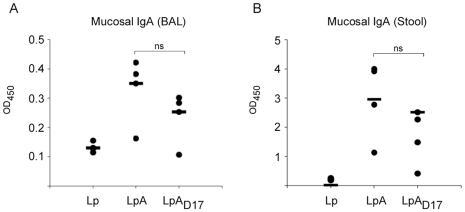
Antibody response to oral administration of recombinant *L. plantarum*: mucosal IgA. C3H-HeJ mice were inoculated intragastrically with *L. plantarum* expressing OspA (LpA) or the mutant OspA_D17_ (LpA_D17_). Control mice were inoculated with *L. plantarum* (Lp). Specific mucosal anti-OspA IgA antibodies were measured by indirect ELISA in broncheoalveolar lavage (BAL) (A) and stool (B) collected on day 68. The results corresponding to each mouse are expressed as Optical Density at 450 nm (OD_450_) of the mean endpoint titer. Results are representative of one of three independent experiments. *n* = 4 mice per group. ns, not significant.

Lastly, to assess the systemic and mucosal antibody immune response induced by the oral administration of recombinant *L. plantarum*, we immunized mice and tested serum levels of OspA-specific total IgG, IgG1 and IgG2a antibodies (Fig. 8), and the levels of mucosal OspA-specifc IgA in bronchoalveolar lavage (BAL) and stool suspensions (Stool) (Fig. 9), by indirect ELISA. In contrast to the controls, mice orally administered with *L. plantarum* expressing lipidated OspA (LpA) or the unlipidated mutant developed high titers of IgG specific antibody 68 days after the first inoculation (Fig. 8A). However, mice inoculated with LpA produced higher levels of OspA-specific IgG1 compared to mice inoculated with LpA_D17_, as shown on day 68 after the first inoculation (**p<0.01) (Fig. 8B). In contrast, mice inoculated with either LpA or LpA_D17_ developed equivalent levels of IgG2a (Fig. 8C). Furthermore, the same mice inoculated with LpA and LpA_D17_ developed a high OspA-specific IgA antibody titer in BAL (Fig. 9A) and stool (Fig. 9B), 68 days after the first inoculation. Differences between LpA and LpA_D17_ were not statistically significant.

## Discussion

It is now generally accepted that mucosal vaccines that can elicit both secretory IgA and effective systemic immune responses could have advantages over many existing vaccines [1]. A large body of research has accumulated recently, whereby mucosal vaccines have been developed using LAB against a vast number of pathogens to various degrees of efficacy success [26], [27], [28], [29], [9], [30], [31], [32], [33], [11], [34]. We have recently found that *L. plantarum* expressing *B. burgdorferi* OspA lipoprotein induced the production of OspA-specific IgA and IgG antibodies as a result of oral administration [12]. In the present study, we investigated the effect of the lipid modification of OspA in the localization of the antigen in our mucosal delivery vehicle, *L. plantarum*. Furthermore, we investigated how recombinant *L. plantarum* expressing OspA lipoprotein breaks the oral tolerance of the gut. We show that the leader peptide of OspA targets the protein to the cell envelope of the delivery vehicle *L. plantarum*, and that the Cys^17^ is the substract for OspA lipidation. Further, we show that our recombinant *L. plantarum* based mucosal delivery system does not induce secretion of IL-8 by intestinal epithelial cells and that it breaks oral tolerance of the gut via Th1/Th2 cell mediated immunity.

Expression of the full-length *B. burgdorferi* OspA gene in *E. coli*, a Gram-negative bacteria, produces a protein that is processed post-translationally by signal peptidase II and contains an attached lipid moiety. In *E. coli* this lipoprotein partitions mostly into the detergent phase following extraction with Triton X-114 [35]. As seen in *E. coli*, we show that in Gram-positive *L. plantarum*, the leader peptide also marks OspA for translocation or export across the cytoplasmic membrane given that wildtype OspA partitions to the detergent phase following extraction with Triton X-114, suggesting that it is lipidated. In contrast, the mutant OspA_D17_, which lacks the cystein that is needed for the lipid moiety attachment, partitions mostly to the aqueous phase. This shift on the solubility of the mutant OspA_D17_ appears to be due to the lack of the lipid attachment, that makes the antigen more hydrophilic than the native lipidated OspA, and therefore the mutant appears to be unlipidated. It appears that OspA is indeed lipidated and localizes mostly at the membrane-cell wall interface. This observation is consistent with a previous study of Gram-positive *Lactococcus* spp. lipoprotein processing that found that lipid modification is predominantly required to retain proteins at the membrane cell-wall interface [36].

The intestinal immune system is the largest and the most complex part of the immune system [23]. Crosstalk occurs between the luminal microorganisms and the mucosal tissue, with intestinal epithelial cells (IECs) mediating this dialogue. Microbial invasion of the gut epithelium stimulates IECs to produce CXC-chemokine ligand 8 (IL-8) and activate other pro-inflammatory pathways that in turn coordinate the innate immune response [37]. The absence of IL-8 secretion by IECs in the presence of recombinant *L. plantarum* is consistent with previous findings [38], [39], [40] and suggests that an oral vaccine based in *L. plantarum* expressing the OspA lipoprotein would not induce the local adverse inflammatory effects attributed to pathogenic bacteria.

In the lamina propria of the intestine, the adaptive immune response is oriented towards tolerance and it involves suppression of Th1 cytokines such as IL-12 and activation of regulatory T cells that inhibit the proliferation of other T cells through production of IL-10 and TGFβ1 [22], [23], [41], [37]. Together with IECs, dendritic cells (DCs) interact with luminal bacteria [37] either through receiving M-cell delivered bacterial cargo or through extension of their own pseudopods across the IEC monolayer [42], [43], [43]. When we stimulated human PBMC-derived-dendritic-cells (PBMC/DCs) with recombinant *L. plantarum* expressing the OspA lipoprotein, but not the unlipidated mutant, we observed that pro-inflammatory cytokines TNFα, IFNγ and IL-6 were markedly upregulated. In addition, we observed an increase of anti-inflammatory cytokine IL-10. When we stimulated monocyte derived dendritic cells (MDDC) with *L. plantarum* expressing lipidated OspA, but not unlipidated OspA, we observed that pro-inflammatory cytokines TNFα and IL-12 were markedly upregulated. Here too, we observed an increase of anti-inflammatory cytokine IL-10. Differences in detection of cytokines in both assays can be explained by the fact that in the former (PBMC/DC) assay we expect to have a mixed population of monocyte derived dendritic cells, T cells, B cells and NK cells, and in the later (MDDC), we expect to have a relatively pure population of dendritic cells. These data indicate that recombinant OspA-expressing-*L. plantarum* stimulates the immune response via Th1/Th2 cell mediated immunity and that, lipidation of the antigen in the delivery agent plays an active role in this response.

The lipid attachment in OspA seems to be a critical determinant of the mature protein immunogenicity [35]. Furthermore, the OspA leader peptide has been fused to different proteins to generate potent lipo-antigens that induce strong B (IgG) and T-cell (helper CD4^+^ and cytotoxic CD8+) responses [44], [45]. Because lipoproteins and OspA are known to trigger TLR2-based receptors [46], [47], we stimulated monocyte derived dendritic cells with purified proteins in order to access the effect of the naked protein on cytokine induction. We observed that wildtype OspA (_wt_OspA) induced TNFα and IL-10 but it did not induce IL-12 or IFNγ. In the absence of the leader peptide, as in _mut_OspA, the TNFα response was abrogated and IL-10 was reduced in half. In contrast, if OspA is delivered in *Lactobacillus* we also observe an effect on the production of IL-12. This effect appears to be related to the delivery vehicle in which OspA is presented. Furthermore, our results show that mutation of the Cys^17^ on the leader peptide of OspA decreases significantly the production of pro- and anti-inflammatory cytokines, and that this effect is related to the lipidation of OspA. In addition, we observed that lipidation of protein is not essential to induce a specific humoral immune response to OspA (systemic IgG and mucosal IgA) when the antigen is orally delivered by *Lactobacillus*. The adjuvant effect of *Lactobacillus* as oral carrier, exerts a synergized effect along with the intrinsic antigenicity of unlipidated OspA. This result differs with others, where the lipid modification of OspA seems to be essential to induce a humoral immune response, when the soluble unlipidated antigen is delivered subcutaneously without a cellular carrier [35]. In addition, we have found that the mutation of OspA Cys^17^ skewed the systemic IgG production towards IgG2a, probably as result of a shift from a Th2 to a Th1 response. Over all, our data suggest that the lipidation of OspA has a clear role on the mixed Th1/Th2 cellular and humoral immune response to orally administered *Lactobacillus* expressing OspA.

Given that we inoculate our animals with very high doses of vaccine for long periods of time our results appear to contradict the dogma that high and repeated doses of stimulating antigen induces oral tolerance [48]. This might be due to the fact that our antigen is delivered via a bacterial system rather than purified protein and therefore the amount that is actually exposed to the mucosal immune system might be much less than the naked antigen.

In summary, we investigated the mechanism by which a mucosal vaccine based in recombinant LAB breaks the immunological tolerance of the gut in order to elicit a protective immune response. It is possible that mucosal tolerance may have been broken by two different mechanisms. In the first scenario, M cells might transport whole recombinant *L. plantarum* or OspA lipoprotein as it is released from lysed recombinant *L. plantarum* across the epithelium where they can induce primary immune responses [1]. In a second scenario, as dendritic cells uptake recombinant LAB and recognized it as the pathogen, the bacteria are no longer retained at the mesenteric lymphnode, as it happens when DCs are loaded with commensal bacteria, but instead migrate to secondary lymphoid tissue and present antigen to T-cells in a systemic environment that is not tolerigenic [49].
